# Diet-Related Buccal Dental Microwear Patterns in Central African Pygmy Foragers and Bantu-Speaking Farmer and Pastoralist Populations

**DOI:** 10.1371/journal.pone.0084804

**Published:** 2013-12-19

**Authors:** Alejandro Romero, Fernando V. Ramírez-Rozzi, Joaquín De Juan, Alejandro Pérez-Pérez

**Affiliations:** 1 Universidad de Alicante, Departamento de Biotecnología, Alicante, Spain; 2 Centre National de la Recherche Scientifique, Unité Propre de Recherche “Dynamique de l’Évolution Humaine,” Paris, France; 3 Universitat de Barcelona, Departament de Biologia Animal, Barcelona, Spain; University of Oxford, United Kingdom

## Abstract

Pygmy hunter-gatherers from Central Africa have shared a network of socioeconomic interactions with non-Pygmy Bantu speakers since agropastoral lifestyle spread across sub-Saharan Africa. Ethnographic studies have reported that their diets differ in consumption of both animal proteins and starch grains. Hunted meat and gathered plant foods, especially underground storage organs (USOs), are dietary staples for pygmies. However, scarce information exists about forager–farmer interaction and the agricultural products used by pygmies. Since the effects of dietary preferences on teeth in modern and past pygmies remain unknown, we explored dietary history through quantitative analysis of buccal microwear on cheek teeth in well-documented Baka pygmies. We then determined if microwear patterns differ among other Pygmy groups (Aka, Mbuti, and Babongo) and between Bantu-speaking farmer and pastoralist populations from past centuries. The buccal dental microwear patterns of Pygmy hunter-gatherers and non-Pygmy Bantu pastoralists show lower scratch densities, indicative of diets more intensively based on nonabrasive foodstuffs, compared with Bantu farmers, who consume larger amounts of grit from stoneground foods. The Baka pygmies showed microwear patterns similar to those of ancient Aka and Mbuti, suggesting that the mechanical properties of their preferred diets have not significantly changed through time. In contrast, Babongo pygmies showed scratch densities and lengths similar to those of the farmers, consistent with sociocultural contacts and genetic factors. Our findings support that buccal microwear patterns predict dietary habits independent of ecological conditions and reflect the abrasive properties of preferred or fallback foods such as USOs, which may have contributed to the dietary specializations of ancient human populations.

## Introduction

Both present-day African Pygmy hunter-gatherers (PHGs), characterized by a reduced adult stature (<160 cm) [1–3], and their non-Pygmy Bantu-speaking neighbors live in the tropical rainforests throughout the Congo Basin in a complex network of economic and social contacts with different subsistence strategies [2,4,5]. In contrast to PHGs, whose lifestyle and culture are forest based, non-Pygmy Bantu speakers have an agro-pastoralist, sedentary lifestyle [1,5–7]. Genetic analyses suggest that gene flow between the two populations is limited and asymmetrical due to sociocultural and demographic constraints [8–11]. Linguistic differences have also been demonstrated [2,12], and phenotypic peculiarities in PHGs are not limited to adult height [13]. Central African rainforest environments show great plant and animal biodiversity [14], including aboveground edible plants and starch-rich plant underground storage organs (USOs), as well as many accessible prey animals. Ethnographic evidence has shown that foraging activities, mainly providing wild yam tubers (*Dioscorea* spp.), supply the bulk of the diet among PHGs, who rarely spend time cultivating plant foods [15–19]. Agricultural resources are obtained mainly by exchange for forest products (meat and honey for iron tools and starchy foods) with Bantu-speaking farmers (BSFs) [20–24]. Reports on dental health [25] and investigations with stable isotopes [26] corroborate ethnographic data that indicate significant dietary differences in animal protein and starch-grain consumption between PHGs and agro-pastoralist Bantu speakers.

Buccal dental microwear, the pattern of microscopic use-wear on nonworking enamel surfaces of premolar and molar teeth, has been shown to reflect the physical properties of chewed foodstuffs and long-term trends in dietary preferences [27]. During food chewing, scratches of different length and orientations are formed across buccal enamel surfaces by the indentation effect of micrometer-scale (10^−6^ m) particles that are harder than enamel, such as plant phytoliths, grit, or quartz dust [28–31]. Thus, the type and amount of chewed abrasives are critical in the formation of tooth wear and microscopic scratch patterns [27,28,31–33]. Distinct buccal microwear patterns have been shown to distinguish nonhuman primates and fossil hominins [34,35], as well as foraging and horticultural populations with distinct dietary habits and food processing methods [36–39]. However, reliable buccal microwear patterns of modern human populations with known diets are still lacking, so the effect of preferred diets and the physical properties of ingested foodstuffs on dental microwear patterns need to be better understood [25,40]. Here we present analyses of buccal microwear patterns of both PHGs and non-Pygmy Bantu-speaking populations from Central Africa with distinct and well-characterized diets, including BSFs, inhabiting the same forest environment as PHGs, and Bantu-speaking pastoralists (BSPs) Maasai populations from savanna habitats [7,14]. We first assessed if the populations’ distinct reported diets (foraging, farmer, and pastoralist subsistence economies) are reflected in buccal microwear patterns. We then analyzed if the differential access to cultivated products among the PHGs was apparent in their buccal microwear patterns, which might provide new insights on the abrasive effect of the consumption of USOs and silica-based aboveground plant foods [34,35,41,42] for the characterization of hominin dietary preferences and adaptations.

## Materials and Methods

### Ethics statement

All participants provided verbal informed consent for this study funded by The French National Research Agency (ANR-11-BSV7-0011). Written consent was not obtained because the Baka people included in the study cannot read or write, and the data were analyzed anonymously. However, the information and acceptance process of all participants was video recorded. A native who spoke French and Baka explained the standard statement to each volunteer. The Ethics Committee of the Centre National de la Recherche Scientifique approves this procedure for illiterate traditional populations. The protocol was in accordance with the Declaration of Helsinki and was approved by the Ethics Commission of the Centre National de la Recherche Scientifique (CNRS-UPR2147) and the Institut National de la Santé et de la Recherche Médicale Paris (INSERM).

### Samples studied

A total of 143 first mandibular molars (M_1_), preferably left side, were analyzed. Teeth from PHGs (*n* = 51) correspond to four different ethnographic groups: extant Baka from Cameroon (*n* = 36) and Aka from Central African Republic (CAR; *n* = 4), Babongo from Gabon (*n* = 6), and Mbuti from the Democratic Republic of Congo (DRC; *n* = 5). The BSFs (*n* = 80) included individuals of different ethnic affiliations from CAR, Congo, DRC, Gabon, and Rwanda, and BSPs (*n* = 12) included a Maasai sample used as a control group. Anthropological and ethnographical data from museum records and available literature were used to assign geographical provenance and dietary habits since no quantitative diet data exist in association with the museum samples we surveyed (see Table S1 in File S1 for sample details).

*In vivo* epoxy casts of Baka Pygmy samples were collected under informed consent in June 2008 at the village Moangé-Le-Bosquet in the Dja Biosphere Reserve (Lomié district, southeastern Cameroon) [3]. The other Pygmy and non-Pygmy samples studied were from skeletal collections at the American Museum of Natural History (New York), the Institut Royal des Sciences Naturelles de Belgique (Brussels), the Musée de l’Homme (Paris), and the University of Geneva (Geneva, Switzerland). 

Individual teeth were classified as PHG and non-Pygmy BSF and BSP using population-based physical [1,3,13], cultural [1,2,12], and genetic criteria [8–11]. All non-Pygmy BSF populations studied were pooled based on similar dietary habits and low levels of phenotypic [3,13] and genetic variation [8–10] within groups compared to PHGs. 

Both PHGs and BSFs inhabited Central African tropical rain forests, whereas the BSP Maasai populations lived in savannah environments [14]. The ecosystem in the Congo Basin is characterized by a closed evergreen forest with vegetation consisting mainly of the evergreen rain forest, semideciduous forest, and mixed forest [14,43,44]. The Central and West African forests extend discontinuously from Senegal in West Africa to extreme western Kenya and northern Angola. The maximum annual rainfall is 1500 mm, and temperatures range from 23.1°C to 25°C. The closed evergreen forest does not show any noticeable seasonal behavior with two rainy seasons (March–June and September–November) and two dry seasons (December–February and July–August) in a year, and faunal and floral species often share similar patterns of distribution [14,43]. 

For the current study no attempt was made to determine individuals’ sex, and only juvenile or adult individuals were selected. Subadult individuals were not considered. Early reports have shown that neither age-related (juvenile and adult age groups) nor sex-related differences in buccal microwear are detectable within farming [36,39] or hunter-gatherer [37] populations. All sample were aged using dental development sequences [45], including Baka individuals, since no accurate age data were available due to the lack of birth records [3]. Individuals presenting fully erupted third lower molar (M_3_) were considered adults. Otherwise, juveniles were limited to individuals with the presence of a fully erupted second lower molar (M_2_) (>15 years ± 36 months). Individuals with no erupted M_2_ were excluded. Marquer [46] provided a detail description of sex and age for the Pygmy sample. When available, the age of non-Pygmy individuals was compared with information provided in the collection data sets. Pygmy and non-Pygmy samples from museums were collected between the middle of 19th century and the first half of the 20th century. Specifically, Pygmy samples used in this study are representative of all Pygmy skulls present in collections, which were deposited between 1879 and 1953 (see Ramírez-Rozzi and Sardi [13] and Marquer [46]). Final Pygmy and non-Pygmy sample sizes correspond to individual skulls with a complete jaw and *in situ* M_1_ teeth suitable for microwear analysis. 

### Pygmy Hunter-Gatherers

Southeastern Cameroon Baka pygmies, one of the largest groups of seminomadic hunter-gatherers in Central Africa, show the lowest genetic admixture rates with BSF neighbors [9,47]. Since they lack both plantations and domestic animals, the Baka pygmies’ subsistence economy heavily depends on forest products [2,19,48]. Over 90% of their daily energy intake on a weight basis is obtained from yam (*Dioscorea* spp.) tuber (60%), game meat from small mammals (15%–20%), and nuts (10%), with few seasonal differences [19]. Other food types (fish, honey, or insects) play a complementary role [19,49]. The Aka pygmies live in the southern forest regions of CAR and northern Congo-Brazaville. Their subsistence is based on gathered foods, mainly yam, and game from hunting. The Aka spend little or no time cultivating plant foods [2,16,22], and consumption of agricultural foods remains very limited [22,50,51]. This pattern of resource accessibility resembles that of the Baka pygmies [2,19,49].

The Mbuti pygmies inhabit the southern part of the Ituri Forest in the DRC. Ethnographic descriptions show that they do not practice agriculture, and hunting activities have been more important than gathering wild vegetables in their subsistence strategies for centuries, with fish representing only a small portion of their diet [4,15]. The Mbuti largely rely on forest products, although they also obtain farm products (cassava, plantain, and agricultural crops) from Bantu villagers [23,24], which does not affect their foraging lifestyle [4,23,52]. Babongo pygmies inhabit forested areas of the central and southern Gabon [53]. Compared with the Aka and Baka, the Babongo people lead a highly sedentary lifestyle [54], having adopted agriculture earlier than other forest peoples but not to an extent sufficient to satisfy all their nutritional requirements [20,54]. At present, meat from the forest occupies an important position in their diet (25%), but starchy foods (cassava, maize, plantains, and peanuts) from farming constitute their major subsistence strategy (46%), which is complemented by plant gathering (13%) and fish (8%) being mainly consumed as a snack [54].

### Bantu-speaking populations

Non-Pygmy BSFs include groups from CAR (Banda, Banziri, Baya, Bayanda, Bopan, Boupara, Mandjia, and Yakoma), Congo (Batéké-Balali and Bondjo), DRC (Azande, Bassoko, Luba, Mamvu, Mayanga, Mongo, and Yombé), Gabon (Adouma, Ashango, Bakalai, Bayaka, Boulou, Bwiti, Galoa, Mpongue, N’Komi, and Pahouin), and Rwanda (Bahutu). All Bantu speakers in this study inhabit the rainforest near or in close contact with pygmies [13]. Ethnographic reports (see references in Table S1 in File S1) show that BSFs tend to cluster in small villages with subsistence strategies mainly dependent on crop plants from small-scale riverine plantations, based upon slash-and-burn techniques [14,15,20,25]. Farmed products include manioc, plantains, maize, rice, and peanuts. Animal farming consists of poultry and some goats, either with or without raising cattle. Despite BSFs occasionally hunting, hunted meat resources are primary obtained from trade with Pygmy groups [2,5,23,25,54]. In contrast to PHGs and BSFs, the BSP Maasai from Kenya and Tanzania have a highly specialized pastoralist diet, mainly based on milk, meat, and blood, with little foraging and no agricultural practices [7].

### Buccal microwear analysis

Tooth crowns were molded with polyvinylsiloxane dental impression material (PresidentJet regular body, Coltène^®^ Corp.) and the resultant high-resolution epoxy casts (Araldite^®^ 2020, Vantico Ltd.) were produced from molds following standard procedures [27,55]. For the *in vivo* Baka sample, prior to silicone-based molds being made, volunteers’ teeth were brushed and dried with an air compressor [22]. Original teeth selected from skeletal collections were cleaned using a cotton swab soaked in pure ethanol, air-dried, and then molded using the dental impression material [55]. All replicas were examined under light microscopy to determine suitability for buccal enamel-surface microwear analysis. Based on standard assessments [33,35,39], casts exhibiting *postmortem* chipping, cracking, or surface erosion on buccal enamel surfaces were discarded. The final sample included only casts that showed buccal enamel surfaces with preserved antemortem microwear features [33,35]. The replicas suitable for buccal microwear analysis were coated with a ~15-nm layer of gold-palladium and analyzed using scanning electron microscopy. Micrographs (1280 × 960 pixels) were taken at 100× magnification in the middle third of the buccal surface of dental crowns, preferably under the protoconid cusp tip, purposely avoiding microwear features caused by intertooth contact on occlusal facets [35]. The digital micrographs were cropped with Adobe Photoshop^TM^ 6.0 to cover exactly 0.56 mm^2^ of the buccal enamel surface and enhanced with a high-pass filter (50 pixel) and automatic level adjustment [35,56]. A total of 10 microwear variables were considered [34], including the scratch density (N) and average length (X) (in micrometers) of all observed lineal scratches ≥10 µm (NT and XT, respectively), which were recorded and measured with Sigma Scan ProV SPSS^TM^ [35,36,56], and eight independent microwear density and length variables, which were classified by 45° orientation intervals (from 0° to 180°) for lower M1 teeth, with regard to the cemento-enamel junction of the tooth, as follows: mesio-distal (NMD, XMD), vertical (NV, XV), horizontal (NH, XH), and disto-mesial (NDM, XDM) [see Pérez-Pérez et al. [36] for detailed variable definitions).

Microwear variables were rank-transformed before running statistical analyses to mitigate effects of noncollinearity of variables distribution and heteroscedasticity [57,58]. Further, ranked data can be used for parametric and multivariate analyses even with small sample sizes [58]. All the variables studied passed Kolmogorov-Smirnov normality tests.

Descriptive statistics and tests at the α = 0.05 significance level were conducted using Addinsoft^TM^ XLSTAT-3.02. A multivariate analysis of variance (MANOVA), followed by one-way analyses of variance (ANOVA) and *post hoc* paired comparisons using Tukey’s Honest Significant Difference test (Tukey’s HSD) were used as needed to check interpopulation differences in microwear patterns. Finally, a Principal Components Analysis (PCA) was done with density-derived (NMD, NV, NH, NDM) and length-derived (XMD, XV, XH, XDM) variables, removing NT and XT variables because their high collinearity levels (*r* Pearson = 0.4 to 0.8; *p* < 0.01) with other variables [39], to show the major trends in buccal microwear among the analyzed groups, as applied previously [35], and to identify the influence of the consumption of abrasive foods on microwear patterns [37].

## Results

**Figure 1:**
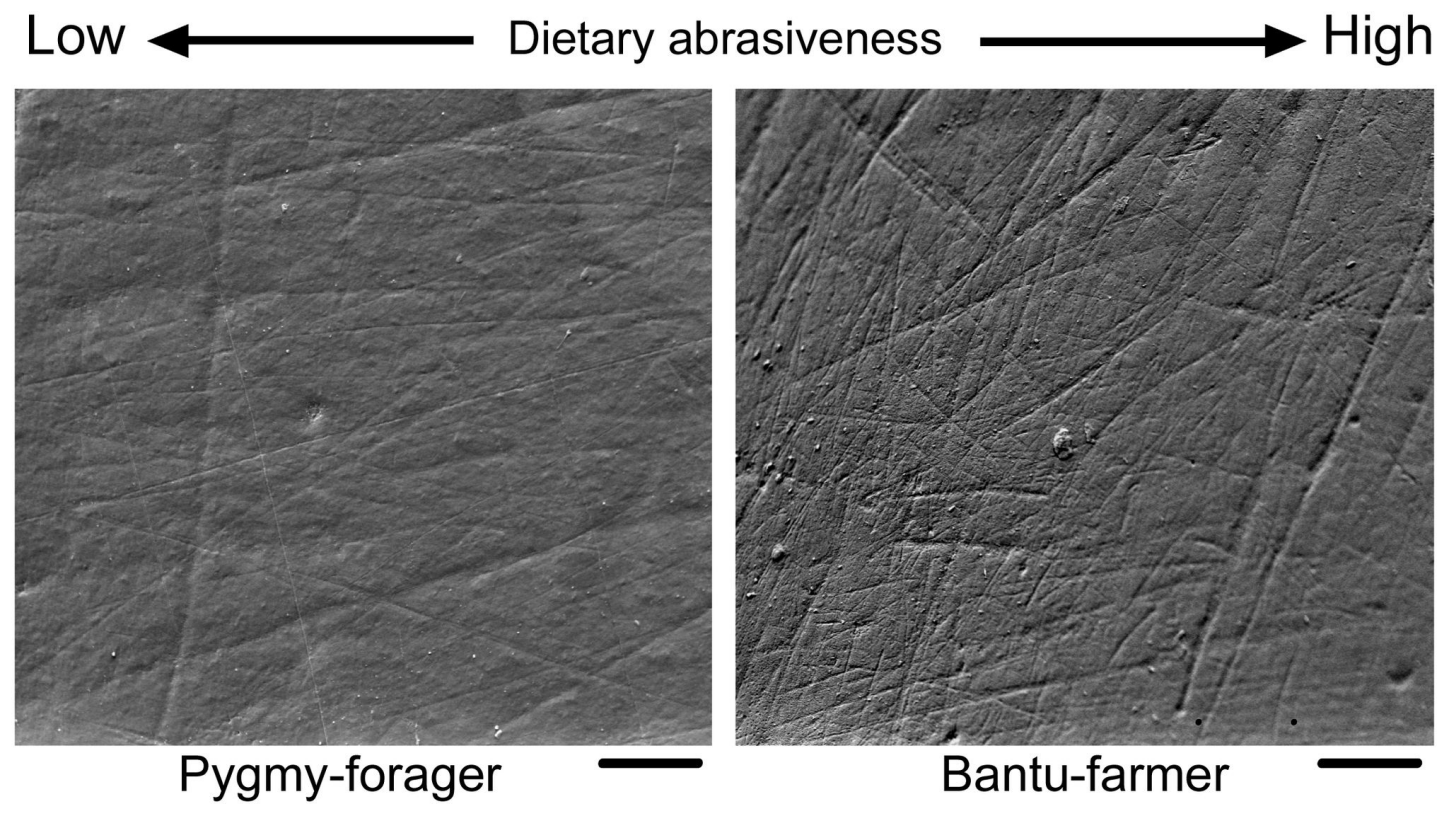
Buccal enamel surfaces showing different microwear patterns related to abrasive properties of chewed foodstuffs. Baka Pygmy hunter-gatherer (left) and Pahouin (#9715, Musée de l'Homme, Paris) agriculturalist from Gabon (right). Both individuals are adult females. Note highly abraded enamel surface in the Bantu-speaking farmer. Each micrograph represents a buccal enamel area of 0.56 mm^2^ on mandibular first molars at 100×. Scale bar: 100 μm.

**Figure 2:**
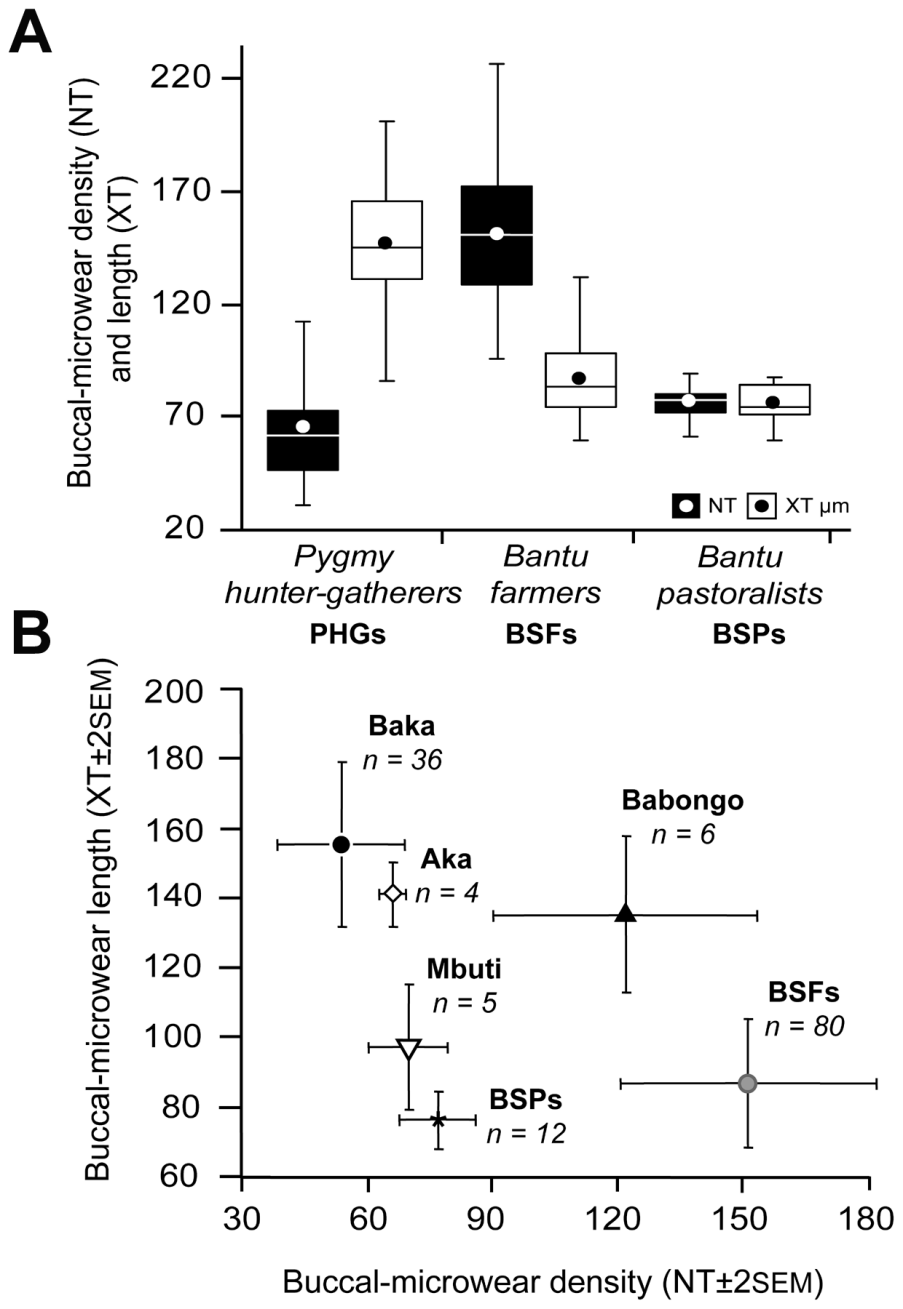
Buccal microwear pattern variability among the analyzed populations. (A) Box-plots showing scratch density (NT) and their average length (XT, in micrometers) among dietary groups. Boxes enclose 25%–75% percentile values, the mean and median are indicated with a circle and horizontal bar, respectively, and whiskers denote minimum–maximum values. (B) NT and XT mean values plotted within PHGs (Aka, Baka, Babongo, and Mbuti) and Bantu farmer (BSFs) and pastoralist (BSPs) populations. Error bars denote ±2 standard error (SEM). The number of individuals (*n*) in each group is indicated.

Overall differences in total scratch densities and average length (in micrometers) were observed between PHGs and Bantu speakers (Figure 1 and Table 1). The PHGs showed the smallest scratch density (NT = 64.47 ± 27.31 –mean ± SD-, *n* = 51), followed by the BSPs (NT = 77.00 ± 9.12, *n* = 12), with the BSFs having the highest scratch density value (NT = 151.25 ± 30.62, *n* = 80). Moreover, the PHGs showed the largest scratch length (XT = 146.37 ± 28.06 μm) (Figure 2A). Within the PHG groups, the Babongo showed the highest density values (NT = 121.83 ± 31.44) and the Mbuti had the lowest XT value (97.37 ± 17.80 μm), close to that of the BSFs (87.11 ± 18.50 μm) (Figure 2B). In an analysis of scratch density and length variables by orientation (see Methods and Table 1), we found that BSFs had higher densities of shorter scratches than the PHGs. Otherwise, the BSPs presented the lowest between-group length values. Among PHGs, the Babongo had the largest scratch densities by orientation, whereas the Mbuti showed the shortest. Significant differences in overall scratch density (NT) and length (XT) were observed among the three main socioeconomic groups: PHGs, BSFs, and BSPs (*F* = 23.44, *p* < 0.001; Wilk’s λ = 0.129, partial ε^2^ = 0.641, MANOVA; Table 2). The univariate ANOVAs showed that among-group differences were significant for all 10 microwear variables (*p* < 0.001). Pairwise comparisons (Tukey’s HSD *post hoc* test) showed (Table 3) that PHGs had significantly (*p* < 0.05) lower scratch densities and larger lengths than the BSFs for all orientations, while the PHGs and BSPs significantly differed for NV and all the length variables, while the BSFs and BSPs differed for all the density variables, except NV, and for XMD and XH (Table 2). The forager group (PHGs), including the Aka, Baka, Babongo, and Mbuti, was characterized by low scratch densities and high scratch lengths, a microwear pattern that was clearly opposite that of the farmer group (BSFs). Although the PHGs showed a more homogenous microwear pattern compared with the Bantu speakers, significant differences were observed among populations when the Baka, Aka, Babongo, and Mbuti were considered separately (Table S2 in File S1). All microwear variables showed significant among-group differences (*p* < 0.001, ANOVA). However, in a comparison of the Aka and Baka (Tukey’s HSD; *p* < 0.05), no significant differences in any microwear variables were found. Instead, the Baka statistically differed from the Mbuti for XT (*p* < 0.001) and XV (*p* = 0.003), and from the Babongo for NT (*p* < 0.001), NMD (*p* = 0.043), and NH (*p* < 0.001), whereas significant differences between the Aka and Babongo were found only for NT (*p* = 0.018). Finally, the Babongo differed from the Mbuti only for NT (*p* = 0.015). The Babongo clearly showed the most distinct microwear pattern among PHGs, resembling the BSFs with regard to high scratch densities. The other PHG groups showed similar scratch densities, but the Mbuti had shorter average scratch lengths, which resulted in a microwear pattern resembling that of the BSPs (Figure 2B)

**Table 1:** Summary statistics of buccal dental microwear pattern for the groups analyzed.

		**Group** ^†^						
**Microwear variables** *		**PHGs (n=51)**	**Baka (n=36)**	**Aka (n=4)**	**Babongo (n=6)**	**Mbuti (n=5)**	**BSFs (n=80)**	**BSPs (n=12)**
**NMD**	Mean	13.86	10.94	12.50	27.66	19.40	39.66	13.41
	SD	11.963	9.096	4.795	19.510	13.011	19.560	8.360
**NV**	Mean	19.09	18.75	19.25	28.00	10.80	28.86	35.16
	SD	11.746	11.517	14.997	11.764	4.024	15.357	10.434
**NH**	Mean	19.41	14.47	20.50	45.33	23.00	53.25	13.08
	SD	15.450	9.476	12.151	25.033	7.713	22.392	4.337
**NDM**	Mean	12.09	9.77	14.00	20.83	16.80	29.47	15.33
	SD	8.164	6.973	6.377	8.232	13.881	15.197	7.819
**NT**	Mean	64.47	53.94	66.25	121.83	70.00	151.25	77.00
	SD	27.313	15.186	3.304	31.447	9.433	30.627	9.125
**XMD**	Mean	111.31	115.48	98.50	116.94	84.51	77.89	61.06
	SD	34.736	37.588	28.014	21.900	16.514	22.870	16.347
**XV**	Mean	177.01	190.35	168.27	156.57	112.53	97.89	86.87
	SD	43.308	38.305	23.413	30.763	38.879	28.916	15.014
**XH**	Mean	138.53	147.16	109.99	138.27	99.48	88.26	61.16
	SD	44.452	44.362	25.098	36.137	44.537	26.326	15.381
**XDM**	Mean	121.27	124.97	152.80	107.64	85.79	74.42	73.21
	SD	38.484	41.437	14.079	11.480	14.344	20.333	17.626
**XT**	Mean	146.37	155.59	141.13	135.41	97.37	87.11	76.33
	SD	28.061	23.563	9.071	22.489	17.806	18.504	8.467

* Data show mean and standard deviation (SD) of the number (N) and length (X) in micrometers of enamel scratches classified in four orientation categories (V, M, D, and H) of 45° intervals and all categories pooled (T). Thus, a total of 10 variables of scratch density (NMD, NV, NH, NDM, and NT) and length (XMD, XV, XH, XDM, and XT) were derived for the sample studied.

^†^ Intergroup division included six groups as follows: four Pygmy hunter-gatherer groups (PHGs), one eastern (Mbuti, *n* = 5) and three western (Baka, *n* = 36; Aka, *n* = 4; Babongo, *n* = 6) Pygmies; one Bantu-speaking farmer group (BSFs, *n* = 80), including geographic dispersed populations from Central African Republic (CAR, *n* = 15), Congo (*n* = 14), Democratic Republic of Congo (DRC, *n* = 22), Gabon (*n* = 21), Rwanda (*n* = 8), and one Bantu-speaking pastoralist (BSP) Maasai (*n* = 12) group.

**Table 2:** Statistical comparisons (MANOVA, ANOVA) between groups by diet.*

	**Value**	**df**	***F***	***p***	**ε^2^**
MANOVA					
Wilk’s λ	0.129	20, 262	23.435	**<0.001**	0.641
Pillai Trace	1.162	20, 264	18.303	**<0.001**	0.581
Hotelling-Lawley Trace	4.518	20, 260	29.368	**<0.001**	0.693
ANOVA					
	NT	2, 140	148.025	**<0.001**	0.679
	XT	2, 140	99.310	**<0.001**	0.587
	NMD	2, 140	51.432	**<0.001**	0.424
	XMD	2, 140	32.634	**<0.001**	0.318
	NV	2, 140	12.800	**<0.001**	0.155
	XV	2, 140	81.462	**<0.001**	0.538
	NH	2, 140	98.229	**<0.001**	0.584
	XH	2, 140	48.750	**<0.001**	0.411
	NDM	2, 140	33.981	**<0.001**	0.327
	XDM	2, 140	42.914	**<0.001**	0.380

* Dietary factor included three dietary categories: hunter-gatherer diet (Baka, Aka, Babongo, and Mbuti Pygmy groups; PHGs *n* = 51); agriculturalist diet (Bantu-speaking farmers, BSFs *n* = 80), and pastoralist diet (Bantu-speaking pastoralist Maasai group, BSPs *n* = 12).

**Table 3:** *Post-hoc* tests within the ANOVA comparisons (Tukey’s HSD) between dietary groups for the scratch density variables.^†^

**NT**	PHGs	BSFs	**XT**	PHGs	BSFs
PHGs	—		PHG	—	
BSFs	**70.912**	—	BSF	**−63.037**	—
BSPs	15.014	**−55.897**	BSP	**−80.250**	−17.212
**NMD**	PHGs	BSFs	**XMD**	PHGs	BSFs
PHGs	—		PHG	—	
BSFs	**54.262**	—	BSF	**−42.566**	—
BSPs	0.889	**53.372**	BSP	**−69.750**	**−27.183**
**NV**	PHGs	BSFs	**XV**	PHGs	BSFs
PHGs	—		PHG	—	
BSFs	**28.283**	—	BSF	**−61.495**	—
BSPs	**51.294**	23.010	BSP	**−72.303**	−10.808
**NH**	PHGs	BSFs	**XH**	PHGs	BSFs
PHGs	—		PHG	—	
BSFs	**61.097**	—	BSF	**−46.517**	—
BSPs	−11.264	**−72.362**	BSP	**−82.897**	**−36.379**
**NDM**	PHGs	BSFs	**XDM**	PHGs	BSFs
PHGs	—		PHG	—	
BSFs	**49.579**	—	BSF	**−52.951**	—
BSPs	13.767	**−35.812**	BSP	**−54.259**	−1.308

^†^ Results show matrices of pairwise mean differences in buccal microwear variables.

Analysis conducted on rank data at *p* < 0.05 (in bold).

**Figure 3:**
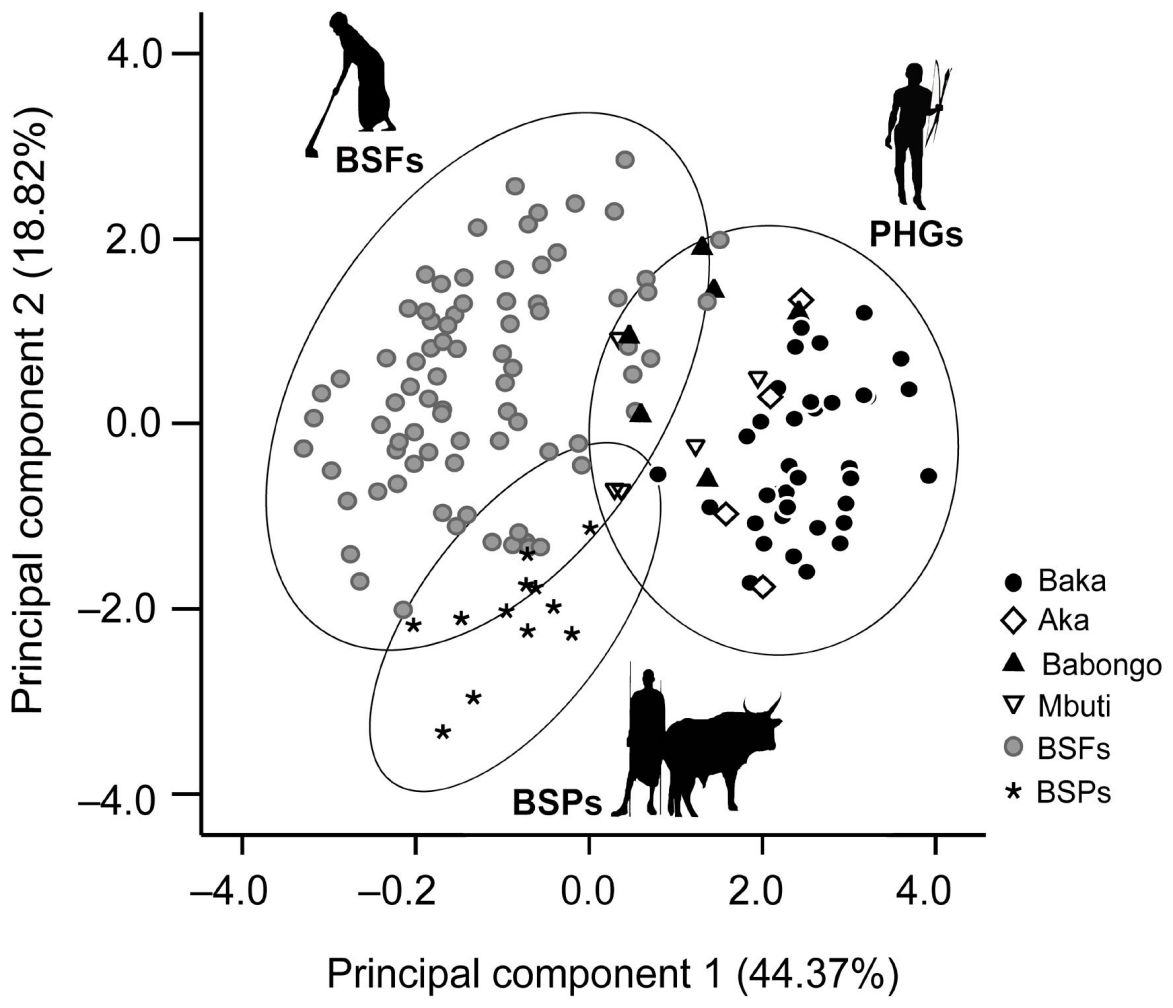
Dispersion Principal Components (PC) 1 versus 2, derived from buccal microwear variables, by subsistence economy. The proportion of the variance explained by the PCs is indicated in parentheses. The ellipses include 95% confidence regions of the dietary groups considered. Pygmy hunter-gatherers (PHGs) have low density of longer scratches, whereas variation in Bantu-speaking farmers (BSFs) and pastoralists (BSPs) are reflected in high (BSFs) and low (BSPs) density pattern of shorter scratches. All individuals analyzed are plotted. See Materials and Methods sections for the details of methodology and sample compositions.

The PCA of the eight independent microwear variables (excluding NT and XT, see Methods) yielded two PCs with Eigenvalues larger than 1 that explained 63.19% of the total variance (Figure 3 and Table S3 in File S1). PC1 explained 44.37% of the total variance and was positively correlated with the length variables (Pearson *r* ranging from 0.72 to 0.83) and negatively correlated with the density ones (*r* values ranging from −0.42 to −0.60). PC2 explained 18.82% of the total variance and was mainly correlated with NH (*r* = 0.69), NMD (*r* = 0.53), and NV (*r* = −0.52). Univariate ANOVAs for PC1 and PC2 by dietary groups (PHGs, BSFs, and BSPs) showed significant differences in both cases (*p* < 0.0001), which indicates that PC1 distinguished PHGs for having longer and less abundant scratches than BSFs and BSPs, and PC2 distinguished BSPs for having a lower scratch density than BSFs. 

## Discussion

Buccal microwear patterns reflect abrasive properties of ingested foodstuffs [36–38], even in present-day living people [33,39]. Meat is not hard enough to damage enamel surfaces during chewing [59], whereas both plant phytoliths and gritty-quartz particles have been shown to cause enamel etching [28,29,31]. However, the effect of specific abrasive agents on enamel surfaces is difficult to characterize because differences in the load-bearing capacity of teeth might be a function of morphology (tooth size and geometry or enamel thickness) rather than underlying mechanical properties [30,32,59,60]. Nonetheless, dental microwear formation seems to be strongly influenced by the amount of abrasive particles ingested with food and their intrinsic hardness compared to enamel [29,31]. Plant silica phytoliths and grit particles have been shown to be involved in the formation and remodeling of microwear patterns [27,29], with phytoliths being responsible for long-term processes, mainly forming small microwear features, and exogenous quartz particles (~2.5-fold harder than enamel) causing significant biting stresses, even with modest loadings, resulting in microwear features larger in size [27,31,32].

The variability of the buccal microwear pattern observed between the BSF and PHG groups was clearly linked to the inclusion of agricultural products in their diets, which likely had a significantly higher content of abrasive particles than foraged fruits and USOs. These microwear patterns are representative of two distinct dietary habits, one mainly vegetarian and including highly abrasive particles and the other having a higher content of hunted meat and foraged plant foods. Otherwise, the two Bantu-speaking groups (BSFs and BSPs) resembled each other in having short scratches; however, the pastoralist Maasai showed a distinct pattern, with both a low density of scratches and short average lengths, likely due to their soft and less abrasive diet [7]. Both PHG and Maasai populations have limited access to cultivated goods (a significant source of phytoliths and dust grit) compared with BSFs [2,4,7,19–21]. Moreover, the PHGs overall showed lower scratch densities and longer average lengths than the BSFs. However, among PHGs the microwear pattern of the Babongo, the most sedentary of the forager populations analyzed, overlapping that of the BSFs, whose samples showed the highest scratch densities and shorter striation lengths. The buccal microwear variability of the Babongo (though represented by a rather small sample) was much smaller than that of the BSFs, showing similarities with the PHG microwear pattern that might suggest they did not fully abandon a forager economy [20]. 

As a whole, the feeding strategies of the PHGs clearly translate into a distinct low-density pattern of buccal microwear that depends on the amount of etching particles incorporated in foodstuffs. Our microwear findings suggest that PHGs mainly consume foraged plant foods with reduced abrasiveness compared to harvested foods. Yam (including over 600 varieties, 95% of which are endemic to Africa), a monocot plant that differs from sweet potatoes (a dicot called “yams” in some parts of the world), is the most consumed starch-rich tuber, either gathered or cultivated, by African populations [41,42], including PHGs [18,19,24,40]. Yam tubers lack phytoliths, useful for taxonomic classification [30], but show the highest levels of fracture toughness (J m^−2^) among 33 analyzed root tubers [42]. If cooked or roasted, their mechanical resistance decreases, while the energetic gain increases, allowing for less forceful mastication and easier digestion [42,61]. If abrasive particles are not incorporated during processing prior to ingestion, yam consumption in rainforest environments does not cause enamel indentation [32,59]. In contrast, USOs from savannah or desert habitats, including a wider range of edible tubers, can be consumed raw [41] and frequently incorporate grit-soil particles that cause higher tooth-wear rates in savannah foragers, such as the Hadzabe in northern Tanzania, than those seen in farmer populations [62]. In PHG culture, animal resources are frequently smoked [4,49,54,63] and tubers, either wild or cultivated, are harvested with a digging stick [18,50] and soaked in water for a few days before being roasted or boiled for consumption [15,19,25,49]. Thereby, both meat and tubers may incorporate abrasive particles from ashes or dust that, despite usually being removed before ingestion, may contribute to the formation of the buccal microwear patterns observed in the PHGs. Otherwise, the highly abraded microwear patterns shown by the Bantu farmers, who consume gritty particles from stoneground foods [5,6], resemble those observed in horticulturalist populations [38,39] and differ from the groups that consume considerable quantities of meat [37]. The consistency of the association seen between food abrasiveness and buccal microwear pattern supports the hypothesis that PHG and BSP populations share an overall soft diet, with significant consumption of meat and other animal-derived products. A diet including some exogenous, coarse, and brittle particles, likely larger in diameter than those in Maasai pastoralist diets, would explain the distinct microwear pattern of the foragers compared with the pastoralist group. 

The increasing reduction of natural areas and scarcity of big game have greatly altered Pygmy economy [5,12,20,21,48]. Manioc cultivation by Baka pygmies was first described at the beginning of the 20th century [64], but it is not yet clear if dietary preferences have changed between ancient and present-day pygmies [2,4,19]. No differences in buccal microwear patterns were observed between the Aka (collected at the beginning of the 20th century) and present-day Baka (collected from living individuals), probably due to similarities in their dietary regimens despite the temporal divergence [2], and few differences were observed between the Baka and the Mbuti (a 1950s collection). The buccal microwear pattern of the Mbuti, and especially that of the Babongo, slightly resembles that of the Bantu farmers, since they engage in food trade contacts [8,9,23,54], whereas the Aka and Baka have less access than the Babongo and Mbuti to cultivated products [2,24,54]. When dietary preferences shift to include greater amounts of abrasives, such as in agricultural goods, buccal enamel microwear patterns have been shown to significantly reflect the change [27,39]. The lack of information about the studied ancient individuals prior to their deaths does not permit seasonal buccal microwear inferences. Nonetheless, a relative importance of wild yam gathering and hunting activities (up to 90%) among the Aka and Baka has been reported in both dry and rainy seasons when no trade for Bantu resources occurs [2,16,19] and supports the homogenous microwear patterns found between these populations. By contrast, we have insufficient data to extend our conclusions to any other PHGs analyzed due to small sample sizes and the scarcity of similar seasonal observational surveys [15,53,54]. Nonetheless, *in vivo* experiments with humans have indicated that buccal-microwear might vary in populations with specific diets and ecological conditions but at the same time, long-term microwear patterns probably remain stable independent of dietary habits [27,33].

Based on overall observation, our results suggest that dietary habits of Pygmy populations have not significantly changed during the last century, as some ethnographic studies have already indicated [4,19,21]. The economic associations between Bantu speakers and Pygmy seem to be limited to specific time periods and vary according to the group, as shown between the Mbuti and Bantu-speaking groups in the Ituri forest [4,12,50,65], and do not significantly affect microwear patterns of most of the forager groups analyzed from Central Africa. Similar information from western Africa is lacking [5,50,51,53]. 

The implications of the buccal microwear variability of the Pygmy and Bantu populations for the characterization of hominin diets should not be dismissed. Meat and USOs may have played a significant role in dietary adaptations of early hominins [41,42] and morphological adaptations of dental traits in the earliest members of the genus *Homo* (2–1.5 Ma) have been related to the consumption of mechanically challenging, tough high-energy foodstuffs, likely associated with brain growth and cultural changes in stone-tool technology [66,67]. However, the USO-eating hypothesis [41,42] on tooth use and microwear has not yet been tested in modern foragers [41: 494]. Our buccal microwear analysis of Pygmy populations indicates that a diet mainly based on fire-processed meat and USOs, similar to that hypothesized for early *Homo* [41,67], should result in a low-density, not highly variable microwear pattern. However, occlusal microwear texture complexity analyses have shown highly variable microwear patterns for this taxon, interpreted as dietary habits characterized by the consumption of non-fracture-resistant foods and low biomechanical demands on chewing [66], consistent with cranio-dental evolutionary changes in early *Homo* [67]. Buccal microwear patterns of early *Homo* specimens, as well as of other modern forager populations, contribute to this microwear interpretation and to the characterization of the significance of staple foods in ancient human diets.

## Supporting Information

File S1 **Table S1, African populations studied by subsistence strategy.** Includes provenance, sample sizes and references. Table **S2**, Between groups statistical comparisons (One-way ANOVA and Tukey’s pots-hoc test) for all the buccal microwear variables considered. Table **S3**, Results (Eigenvalues, % of explained variance, and Pearson correlations *r*) of the Principal Components (PCA) on buccal dental microwear patterns for the populations considered.
(DOCX)
