# Supplementary material for: Diet-Related Buccal Dental Microwear Patterns in Central African Pygmy Foragers and Bantu-Speaking Farmer and Pastoralist Populations
Source: PLoS One. 2013 Dec 19;8(12):e84804. doi: 10.1371/journal.pone.0084804 (PMC3868657; doi:10.1371/journal.pone.0084804)
Supplement: File S1 — Table S1, African populations studied by subsistence strategy. Includes provenance, sample sizes and references. Table S2, Between groups statistical comparisons (One-way ANOVA and Tukey’s pots-hoc test) for all the buccal microwear variables considered. Table S3, Results (Eigenvalues, % of explained variance, and Pearson correlations r) of the Principal Components (PCA) on buccal dental microwear patterns for the populations considered. (DOCX) [file pone.0084804.s001.docx]

**Supporting Information**

**Table S1.** African populations studied by subsistence strategy. Includes provenance, sample sizes, and references.

| **Population** | **Geographic location** | **Country** | ***n*** | **Sample origin*** | **Source** |
| --- | --- | --- | --- | --- | --- |
| Pygmy hunter-gatherers (PHGs) |  |  |  |  |  |
| Aka | M’bueto | CAR | 4 | MH | [1] |
| Baka | Lomié | Cameroon | 36 | *In vivo* | [2–4] |
| Babongo | Zanaga | Gabon | 6 | MH | [1, 5, 6] |
| Mbuti | Ituri | DRC | 5 | UG | [1, 7] |
| Bantu-speaking farmers (BSFs) |  |  |  |  |  |
| Banda | Sibut | CAR | 1 | MH | [8] |
| Banziri | Sangha | CAR | 1 | MH | [9] |
| Baya | Mambéré-Kadéï | CAR | 1 | MH | [10] |
| Bayanda | Mambéré-Kadéï | CAR | 3 | MH | [11] |
| Bopan | Mambéré-Kadéï | CAR | 3 | MH | [11] |
| Boupara | Mambéré-Kadéï | CAR | 2 | MH | [11] |
| Mandjia | Lobaye | CAR | 1 | MH | [12] |
| Yakoma | Haut Oubangui | CAR | 3 | MH | [11] |
| Batéké-Balali | Batéké Plateau | Congo | 10 | MH | [13] |
| Bondjo | Likouala | Congo | 4 | MH | [14] |
| Azande | Uele | DRC | 4 | IRScNB | [15, 16] |
| Bassoko | Kisangani | DRC | 2 | IRScNB | [16] |
| Luba | Kananga | DRC | 7 | IRScNB | [11, 17] |
| Mamvu | Ituri | DRC | 1 | MH | [18] |
| Mayanga | Dungu | DRC | 1 | IRScNB | [16] |
| Mongo | Kasai river | DRC | 4 | IRScNB | [16, 19] |
| Yombé | Bandundu | DRC | 3 | IRScNB, MH | [20] |
| Adouma | Lastoursville | Gabon | 2 | MH | [12,16, 21] |
| Ashango | N'Gounie | Gabon | 1 | MH | [12,16, 22] |
| Bakalai | Ogooué-Lolo | Gabon | 4 | MH | [12, 23] |
| Bayaka | Nyanga | Gabon | 2 | MH | [11] |
| Boulou | Komo-Mondah | Gabon | 1 | MH | [12, 24, 25] |
| Bwiti | Mayumba | Gabon | 1 | MH | [11, 25] |
| Galoa | Lamberené | Gabon | 1 | MH | [24, 26] |
| Mpongue | Libreville | Gabon | 1 | MH | [16, 21, 26, 27] |
| N’Komi | Fernan Vaz | Gabon | 1 | MH | [26, 28] |
| Pahouin | Moyen-Ogooué | Gabon | 7 | MH | [24, 25, 26] |
| Bahutu | Lake Kivu | Rwanda | 8 | IRScNB | [29] |
| Bantu-pastoralists (BSPs) |  |  |  |  |  |
| Maasai | NW Nairobi/Lake Tanganyika | Kenya/Tanzania | 12 | AMNH, MH | [30] |

*All samples refer to replicas of mandibular M1 teeth (one tooth per individual). With the exception of Baka PHGs *in vivo* sample (fieldwork in Cameroon 2008), replicas were obtained from original *in situ* teeth molded and housed at the AMNH: American Museum Natural History (New York); IRScNB: Institut Royal des Sciences Naturelles de Belgique (Brussels); MH: Musée de l’Homme (Paris) and UG: University of Geneva (Geneva, Switzerland). Population term and geographic origin were obtained from Museum record.

**References**

1. Marquer P (1972) Nouvelle contribution à l’étude du squelette des pygmées occidentaux du centre africain comparé à celui des pygmées orientaux. Paris: Mémoires du Musée National d’Histoire Naturelle. série A, LXXII. 122 p.
2. Vallois HV, Marquer P (1976) Les pygmées Baka du Cameroun: anthropologie et ethnographie avec une annexe démographique. Paris: Mémoires du Musée National d’Histoire Naturelle. série A, C. 195 p.
3. Crampel P (1890) Les Bayagas, petits hommes de la grande forêt équatoriale-lettre à Harry Alis. Compte Rendu des S’eances de la Société de G’eographie 16-17: 548–554.
4. Lalouel DJ (1950) Les Babinga du Bas-Oubangui. Contribution à l’étude anthropologique des Négrilles Baka et Bayaka. Bull et Mém Soc Anthropol Paris. Série 1: 60–98.
5. Marche A (1877) Lettres de l’Ogooué. Bulletin de la Société de Géographie 14: 393–404.
6. Santesson C (1939) Die Babongo-Zwerge und ihr Pfeilgift. Etnologiska studier 8: 137–148.
7. Bakonyi M (1976) Contribution à l'étude du squelette des pygmées (ba)Mbuti de l'Ituri (Zaïre). Thèse, Université de Genève. 60 p.
8. Daigre J (1931) Les Bandas de l'Oubangui-Chari (Afrique Equatoriale Française). Antropos 26: 647–695.
9. Clozel MFJ (1896) The Banziris of the Congo basin. Popular Science 49: 673–677.
10. Burnham P (1984) Gbaya. In: Weekes RV, editor. Muslim peoples: a world ethnographic survey. Wesport Greenwood Press. pp. 286–290.
11. Olson JS (1996) The peoples of Africa. An ethnohistorical dictionary. Greenwood: Westport Ct. 681 p.
12. Poutrin L (1910) Contribution à l'étude des Pygmées d'Afrique. Les Négrilles du Centre africain (type brachycéphale). L'Anthropologie 21: 435–504.
13. Trezenem E (1940) Contribution à l’étude des nègres africains: Les Bateke Balali. Journal de la Société des Africanistes 10: 1–63.
14. Samarin WJ (1984) Bondjo ethnicity and colonial imagination. Canadian Journal of African Studies 18: 345–365.
15. Evans-Pritchard EE (1937) Witchcraft, Oracles and Magic among the Azande. Oxford: Claredon Press. 558 p.
16. Ribot I (2011) A study through skull morphology on the diversity of Holocene African populations in a historical perspective. BAR Int Ser 2215. Oxford: Archaeopress. 217 p.
17. Ehret C (2002) The civilizations of Africa: a history to 1800. Charlottesville: University Press of Virginia. 480 p.
18. Van Geluwe H (1957) Mamvu-Mangutu et Balese Mvuba. London: International African Institute*.* 195 p.
19. Kerken G (1944) L’Ethnic Mongo*.* Bruxelles.
20. Overbergh C, Édouard J (1907) Les Mayombe (État indépendant du Congo). Bruxelles. 470 p.
21. Gardinier DE (1994) Historical Dictionary of Gabon. Oxford: The Scarecrow Press. 455 p.
22. Du Chaillu P (1863) Voyages et Adventures dans l’Afrique equatoriale. Paris: Levy. 547 p.
23. Hugh C (1911) Encyclopædia Britannica. Cambridge: Cambridge University Press. 11th ed. Volume 3.
24. Liotard M (1895) Les races de l’Ogooué. L’Anthropologie 6: 53–64.
25. Alexandre O, Binet J (1958). Le groupe dit Pahouin (Fang. Boulou. Béti). Paris: Coll Monographies ethnologiques africaines de l'Institut International Africain. 152 p.
26. Haug E (1903) Le Bas-Ogooué. Annales de géographie 62: 159–171.
27. Bucher HH (1975) Mpongwe origins: historiographical perspectives. History in Africa 2: 59-89.
28. Benington RC (1912) A study of the negro skull with special reference to the Congo and Gaboon crania. Biometrika 8: 292–339.
29. Brabant H (1963) Observations anthropologiques et odontologiques sur les dents des Hutu du Rwanda. Annales du Musée Royal de l’Afrique Centrale 47: 1–30.
30. Reinecke P (1896) Beschreibung einiger rassenskelette aus Afrika. Archiv für Anthropologie 25: 185–227.

**Table S2.** Between groups statistical comparisons (One-way ANOVA and Tukey’s *pots-hoc* test) for all the buccal microwear variables considered.

|  |  |  |  |  |  |
| --- | --- | --- | --- | --- | --- |
| **ANOVA** | **Variable** | **df1** | **df2** | **F** | ***p*** |
|  | NT | 5 | 137 | 83.828 | **<0.001** |
|  | XT | 5 | 137 | 49.311 | **<0.001** |
|  | NMD | 5 | 137 | 23.494 | **<0.001** |
|  | XMD | 5 | 137 | 14.071 | **<0.001** |
|  | NV | 5 | 137 | 6.696 | **<0.001** |
|  | XV | 5 | 137 | 38.565 | **<0.001** |
|  | NH | 5 | 137 | 51.961 | **<0.001** |
|  | XH | 5 | 137 | 22.089 | **<0.001** |
|  | NDM | 5 | 137 | 16.117 | **<0.001** |
|  | XDM | 5 | 137 | 18.850 | **<0.001** |
|  |  |  |  |  |  |
| **Tukey’s HSD pairwise comparison test^†^** | | | | | |
|  |  |  |  |  |  |
| **NT** | Baka | Aka | Babongo | Mbuti | BSF |
| Baka | - |  |  |  |  |
| Aka | 14.930 | - |  |  |  |
| Babongo | **58.763** | **43.833** | - |  |  |
| Mbuti | 16.980 | 2.050 | **−41.783** | - |  |
| BSF | **80.661** | **65.731** | 21.897 | **63.681** | **-** |
| BSP | **24.763** | 9.833 | **−34.000** | 7.783 | **−55.897** |
|  |  |  |  |  |  |
| **XT** | Baka | Aka | Babongo | Mbuti | BSF |
| Baka | - |  |  |  |  |
| Aka | −10.027 | - |  |  |  |
| Babongo | −13.277 | −3.250 | - |  |  |
| Mbuti | **−55.377** | −45.350 | −42.100 | - |  |
| BSF | **−70.815** | **−60.787** | **57.537** | −15.437 | - |
| BSP | **−88.027** | **−78.000** | **−74.750** | −32.650 | −17.212 |
|  |  |  |  |  |  |
| **NMD** | Baka | Aka | Babongo | Mbuti | BSF |
| Baka | - |  |  |  |  |
| Aka | 8.319 | - |  |  |  |
| Babongo | **40.152** | 31.833 | - |  |  |
| Mbuti | 20.519 | 12.200 | −19.633 | - |  |
| BSF | **61.650** | **53.331** | 21.497 | 41.131 | - |
| BSP | 8.277 | −0.041 | −31.875 | −12.241 | **−53.372** |
|  |  |  |  |  |  |
| **XMD** | Baka | Aka | Babongo | Mbuti | BSF |
| Baka | - |  |  |  |  |
| Aka | −11.861 | - |  |  |  |
| Babongo | 9.805 | 21.666 | - |  |  |
| Mbuti | −29.761 | −17.900 | −39.566 | - |  |
| BSF | **−45.261** | −33.400 | **−55.066** | −15.500 | - |
| BSP | **−72.444** | **−60.583** | **−82.250** | −42.683 | −27.183 |
|  |  |  |  |  |  |

**Table S2.** Cont.

| **Tukey’s HSD pairwise comparison test^†^** | | | | | |
| --- | --- | --- | --- | --- | --- |
|  |  |  |  |  |  |
| **NV** | Baka | Aka | Babongo | Mbuti | BSF |
| Baka | - |  |  |  |  |
| Aka | 3.222 | - |  |  |  |
| Babongo | 31.472 | 28.250 | - |  |  |
| Mbuti | −29.177 | −32.400 | −60.650 | - |  |
| BSF | **29.378** | 26.156 | −2.093 | **58.556** | - |
| BSP | **52.388** | 49.166 | 20.916 | **81.566** | 23.010 |
|  |  |  |  |  |  |
| **XV** | Baka | Aka | Babongo | Mbuti | BSF |
| Baka | - |  |  |  |  |
| Aka | −8.944 | - |  |  |  |
| Babongo | −16.111 | −7.166 | - |  |  |
| Mbuti | **−49.744** | −40.800 | −33.633 | - |  |
| BSF | **−68.969** | **−60.025** | **−52.858** | −19.225 | - |
| BSP | **−79.777** | **−70.833** | **−63.666** | −30.033 | −10.808 |
|  |  |  |  |  |  |
| **NH** | Baka | Aka | Babongo | Mbuti | BSF |
| Baka | - |  |  |  |  |
| Aka | 12.486 | - |  |  |  |
| Babongo | **56.777** | 44.291 | - |  |  |
| Mbuti | 20.061 | 7.575 | −36.716 | - |  |
| BSF | **70.723** | **58.237** | 13.945 | **50.662** | - |
| BSP | −1.638 | −14.125 | **−58.416** | −21.700 | **−72.362** |
|  |  |  |  |  |  |
| **XH** | Baka | Aka | Babongo | Mbuti | BSF |
| Baka | - |  |  |  |  |
| Aka | −23.166 | - |  |  |  |
| Babongo | −0.916 | 22.250 | - |  |  |
| Mbuti | −40.916 | −17.750 | −40.000 | - |  |
| BSF | **−52.454** | −29.287 | **−51.537** | −11.537 | - |
| BSP | **−88.833** | **−65.666** | **−87.916** | −47.916 | **−36.379** |
|  |  |  |  |  |  |
| **NDM** | Baka | Aka | Babongo | Mbuti | BSF |
| Baka | - |  |  |  |  |
| Aka | 18.652 | - |  |  |  |
| Babongo | 41.277 | 22.625 | - |  |  |
| Mbuti | 23.627 | 4.975 | −17.650 | - |  |
| BSF | **58.215** | 39.562 | 16.937 | 34.587 | - |
| BSP | 22.402 | 3.750 | −18.875 | −1.225 | **−35.812** |
|  |  |  |  |  |  |
| **NDM** | Baka | Aka | Babongo | Mbuti | BSF |
| Baka | - |  |  |  |  |
| Aka | 24.111 | - |  |  |  |
| Babongo | 0.527 | −23.583 | - |  |  |
| Mbuti | −29.738 | −53.850 | −30.266 | - |  |
| BSF | **−53.913** | **−78.025** | **−54.441** | −24.175 | - |
| BSP | **−55.222** | **−79.333** | **−55.750** | −25.483 | −1.308 |

*Intergroup division included six groups (see Table 1 for details).

^†^Results show matrices of pairwise mean differences in buccal microwear variables.

Analysis conducted on rank data at *P* < 0.05 (in bold).

**Table S3.** Results (Eigenvalues and correlations *r*) of the Principal Components (PCA)* on buccal dental microwear patterns for the populations considered.

| **Components** | **1** | **2** | **3** |
| --- | --- | --- | --- |
| Eigenvalues | 3.549 | 1.506 | 0.916 |
| % variance | 44.373 | 18.826 | 11.459 |
| **Variables** | ***r*** | ***r*** | ***r*** |
| NM | −0.540 | 0.526 | 0.557 |
| NV | −0.421 | −0.522 | 0.486 |
| NH | −0.596 | 0.693 | −0.001 |
| ND | −0.572 | 0.305 | −0.504 |
| XM | 0.721 | 0.415 | 0.275 |
| XV | 0.830 | −0.040 | 0.131 |
| XH | 0.743 | 0.450 | 0.033 |
| XD | 0.796 | 0.064 | −0.145 |

*PCA included six groups and 10 buccal microwear density and length (in μm) variables (see Table 1 for groups and variable description details).
